# Unraveling the relationship between childhood dry eye symptoms and sleep patterns

**DOI:** 10.1016/j.jped.2025.101471

**Published:** 2025-11-11

**Authors:** Qing He, Ziwen Sun, Ruixin Li, Yanling Wang, Haoru Li, Desheng Song, Bei Du, Lin Liu, Ruihua Wei

**Affiliations:** a Tianjin Key Laboratory of Retinal Functions and Diseases, Tianjin Branch of National Clinical Research Center for Ocular Disease, Eye Institute and School of Optometry, Tianjin Medical University Eye Hospital, Tianjin, China; b Eye Institute of Shandong First Medical University, Qingdao Eye Hospital of Shandong First Medical University, State Key Laboratory Cultivation Base, Shandong Provincial Key Laboratory of Ophthalmology, School of Ophthalmology, Shandong First Medical University, Qingdao, China; c China Assistive Devices and Technology Center for Persons with Disabilities, Beijing, China

**Keywords:** Dry eye, Children, Sleep, 5-item dry eye questionnaire, Children's sleep habits questionnaire

## Abstract

**Objective:**

Given the increasing incidence of dry eye in children and the established role of sleep as a key health determinant, this study aimed to examine the prevalence of dry eye in children and its association with sleep, adjusting for confounders.

**Method:**

This study included 169,080 children aged 6–12 years. Dry eye syndromes (DEs) were assessed using the 5-Item Dry Eye Questionnaire, whereas sleep quality was evaluated using the Children's Sleep Habits Questionnaire. Logistic and linear regression analyses were performed.

**Results:**

Overall, 13.12% of the children exhibited DEs, and 66.25% experienced poor sleep. Children with poor sleep quality had a significantly higher prevalence of DEs (15.97%, 17,889/112,018) than those with normal sleep quality (7.53%, 4296/57,062) (*P* < 0.001). The prevalence of poor sleep was 80.64% (17,889/22,185) in children with DEs, compared to 64.08% (94,129/146,895) in those without DEs (*P* < 0.001). After adjusting for age, sex, body mass index, and other risk factors, poor sleepers had a higher risk of developing DEs than good sleepers (odds ratio [OR] = 2.005; 95% confidence interval [CI]: 1.933–2.080). Children who slept for < 10 h were more likely to have DEs (OR = 1.236l; 95% CI: 1.167–1.310). In logistic regression analyses stratified by age and sex, poor sleepers showed a high risk of DEs. Moreover, the three dry eye symptoms and sleep were related (*P* < 0.001).

**Conclusions:**

This large-scale study revealed that all domains of sleep were significantly poorer in participants with DEs in children, and these associations remained significant after adjusting for comorbidities.

## Introduction

Dry eye (DE) is a prevalent ocular surface disease marked by the disruption of tear film homeostasis alongside ocular symptoms. The global prevalence of DE ranges from 5 % to 50 % [1,2]. Recent studies suggest that the incidence of DE may increase among children and adolescents. The treatment of DE is associated with a high economic burden for systems and individuals, with annual costs of management estimated at > $50 billion in the United States [3]. Consequently, identifying risk factors for DE is important, as it may help improve interventions and outcomes. Some evidence suggests that lifestyle-related DE may be a distinct disease type, associated with factors such as use of video display terminals, limited outdoor activities, vitamin A deficiency, and poor sleep.

Sleep problems in children are a global public health concern. Adequate sleep is required for satisfactory child development, and behavioral, cognitive, and emotional regulation, as well as physical health [4]. The American Academy of Sleep Medicine and Sleep Research expert consensus recommends that adults and adolescents should sleep 7–9 or even more hours per night [5]. Several studies have investigated the relationship between sleep quality and adult DE, [6,7] reporting that 25.9 %–81.7 % of adults with this condition have trouble sleeping. However, the relationship between DE and sleep in children has not been evaluated yet. This study aimed to investigate the prevalence of DE syndromes (DEs) in children and assess the connection between DEs and sleep while accounting for potential confounding factors. Additionally, the authors examined the correlation between sleep duration and DEs. Finally, this study explored the association between DEs dimensions and sleep.

## Method

This observational cross-sectional study recruited children from Tianjin, China, between January 2023 and May 2023. In total, 172,429 children between the ages of 6 and 12 years participated in this study and completed the questionnaire. Individuals with significant ocular infections or inflammation in the previous month, with ocular trauma, chemical injury to the cornea and conjunctiva, and incomplete questionnaires, were excluded from the study. Finally, the authors obtained 169,080 questionnaires. The questionnaire-based data collection for this study was conducted utilizing an online platform. The survey link was officially disseminated by the Tianjin Municipal Education Commission to schools, with teachers providing necessary assistance to guide students and parents through the completion process. All questionnaires, which included information on demographic characteristics, were collected with consent from the participants’ parents or guardians. The Personal Information and Lifestyle Habits Questionnaire was completed by the parents or guardians of the participating children. The Dry Eye Questionnaire (DEQ-5), which assesses subjective experiences, was self-reported by the children. For younger children or those with comprehension difficulties, parents provided assistance by reading the questions in a neutral and non-leading manner. The Children's Sleep Habits Questionnaire (CSHQ) was self-reported by the parents; parents were asked to recall sleep behaviors occurring over a “typical” recent week. All questionnaires can be accessed in Nthe Supplementary Materials. This study was approved by the Medical Ethics Committee of the Tianjin Medical University Eye Hospital (ChiCTR2200065710). The procedures used in this study adhered to the tenets of the Declaration of Helsinki.

### Assessment of personal information and lifestyle habits questionnaire

This study gathered data on the participants' demographic and clinical characteristics, including age, sex, body mass index (BMI), recent eye surgery history, myopia or hyperopia status, contact lens usage, duration of contact lens wear, daily electronic device usage time, gaming time, e-book reading and Internet browsing time, television viewing time, outdoor exercise duration, and presence of anorexic or food-paranoid tendencies. All variables were adjusted for as potential confounding factors.

### Assessment of dry eye syndromes

In this study, the authors used the 5-Item Dry Eye Questionnaire (DEQ-5) to assess DE syndromes (DEs). Research establishes that there are currently no universally accepted diagnostic criteria for pediatric dry eye, especially in children aged 6 to 15, [8] but the DEQ-5 is considered suitable for this purpose to describe DEs [9]. The DEQ-5 consists of five questions to assess three symptoms, as follows: eye discomfort, eye dryness, and watery eyes, with the frequency of symptoms assessed by five items on a scale from 0 to 4, and the severity of symptoms assessed by six items on a scale from 0 to 5. A total score greater than ≥ 6 for the five questions was considered indicative of DEs. In this study, the Cronbach alpha of the questionnaire was 0.878.

### Assessment of sleep quality

This study used the Children's Sleep Habits Questionnaire (CSHQ) to assess the participants' sleep quality over the previous month. Owens developed the questionnaire for children aged 4–12 years [10]. The questionnaire reflects eight dimensions of common sleep problems in children, as follows: bedtime resistance, sleep-onset delay, sleep duration, sleep anxiety, night wakings, parasomnias, sleep-disordered breathing, and daytime sleepiness. A total score of > 41 indicates poor sleep. The Chinese version has been shown to have good reliability and validity. In this study, the Cronbach alpha of the questionnaire was 0.726.

### Statistical analysis

Statistical analysis was performed using SPSS software (version 23.0, IBM Corp., Armonk, NY, USA). Means and standard deviations were used to describe continuous variables. The independent sample *t*-test and the χ^2^ test were used for comparisons between the continuous and categorical variables, respectively. Logistic regression models were used to analyze eight dimensions of sleep and DE. These models were adjusted for age, sex, BMI, and some confounding factors. Age and sex were stratified. This study aimed to determine whether the relationship between DE and sleep was affected by age and sex. Linear regression analyses were used to assess the correlations between the DEQ-5 and CSHQ dimensions. All tests were two-sided, and statistical significance was set at P-values of < 0.05.

## Results

A total of 169,080 children with a mean age of 9.37(1.71) years were included in this study, of which 88,003 (52.05 %) were males and 81,077 (47.95 %) were females. Of these, 13.12 % had DEs, and 66.25 % had poor sleep (Table 1). In addition, the prevalence of DEs was 13.88 % in males and 12.30 % in females (χ_2_ = 93.07, *P* < 0.001). The prevalence of poor sleep was 65.79 % in males and 66.75 % in females (χ_2_ = 17.495; *P* < 0.001).

**Table 1:** General characteristics of the study population.

	All participants (*n* = 169,080)
Age (Mean(SD))	9.3(1.71)
Male/female	88,003(52.05 %)/81,077(47.95 %)
BMI	19.47(6.06)
Dry eye (DEQ-5)	2.98(3.06)
DEQ ≥ 6	22,185(13.12 %)
Poor sleep(CSHQ > 41)	112,018(66.25 %)
Bedtime resistance	79,199(46.84 %)
Sleep onset delay	109,411(64.71 %)
Sleep duration	130,893(77.41 %)
Sleep anxiety	83,523(49.40 %)
Night wakings	29,073(17.19 %)
Parasomnias	53,770(31.80 %)
Sleep disordered breathing	16,930(10.01 %)
Daytime sleepiness	154,801(91.55 %)

**Fig. 1:**
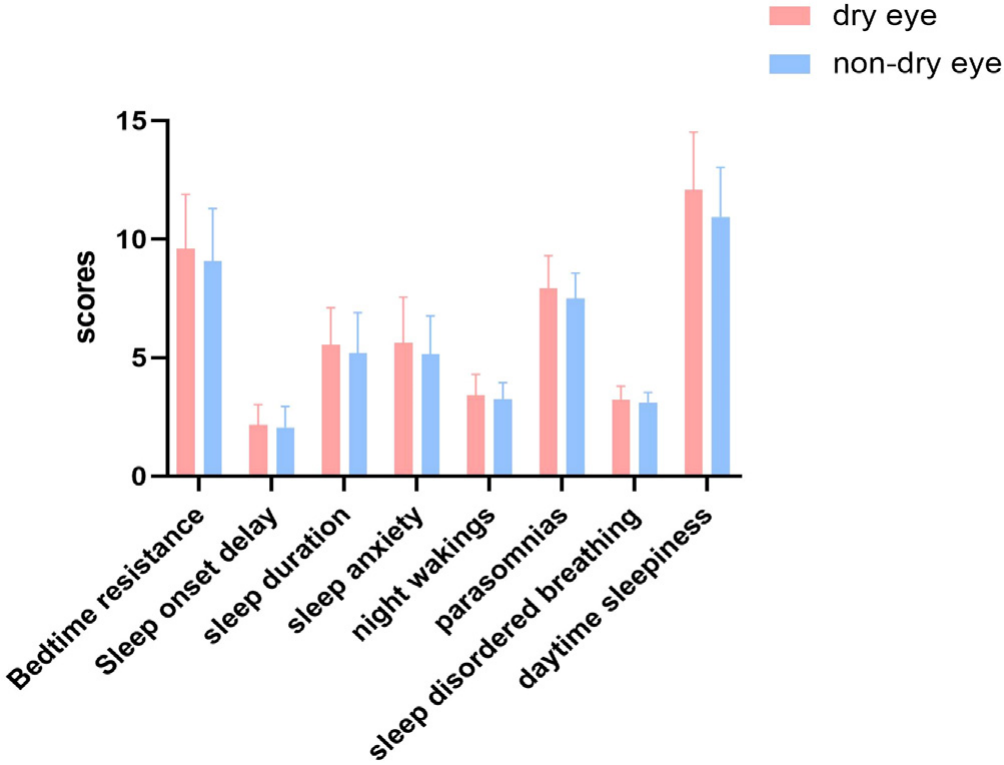
Comparison of sleep parameters in dry eye and non-dry eye patients.

Sleep quality was poorer in children with DEs than in those without DEs (Table 2). In addition, children with DEs scored significantly higher than those without DEs on all eight sleep scores (Figure 1).

**Table 2:** Comparison of sleep parameters in dry eye syndromes (DEs) and non-dry eye syndromes (Non-DEs) patients.

	DEs group	Non-DEs group	t	*P*
N	22,185	146,895		
Total CSHQ score	46.38(5.82)	43.41(5.65)	72.76	**<0.001**
Bedtime resistance	9.60(2.29)	9.07(2.22)	32.72	**<0.001**
Sleep onset delay	2.17(0.85)	2.06(0.88)	16.34	**<0.001**
Sleep duration	5.56(1.55)	5.19(1.71)	30.51	**<0.001**
Sleep anxiety	5.63(1.92)	5.14(1.63)	40.40	**<0.001**
Night wakings	3.42(0.87)	3.26(0.69)	31.45	**<0.001**
Parasomnias	7.92(1.38)	7.50(1.07)	51.56	**<0.001**
Sleep disordered breathing	3.23(0.57)	3.11(0.42)	36.69	**<0.001**
Daytime sleepiness	12.08(2.44)	10.95(2.08)	73.60	**<0.001**

**Fig. 2:**
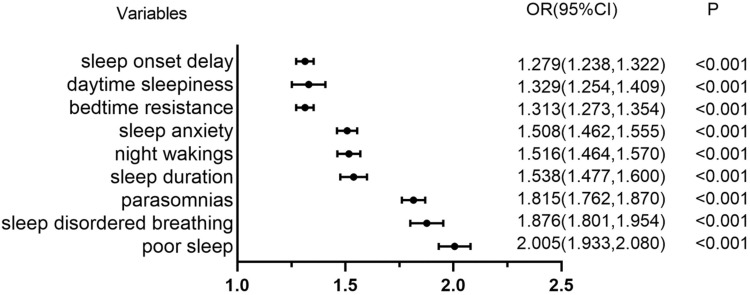
Logistic regression analysis between sleep components and dry eye.

Children with poor sleep quality had a significantly higher prevalence of DEs (15.97 %, 17,889/112,018) than those with normal sleep quality (7.53 %, 4296/57,062) (*P* < 0.001). The prevalence of poor sleep was 80.64 % (17,889/22,185) in children with DES, compared to 64.08 % (94,129/146,895) in those without DEs (*P* < 0.001). The logistic regression results showed that the risk of developing DEs was higher in children with poor sleep than in those with good sleep (Table 3). After adjusting for age, sex, BMI, and other risk factors, poor sleepers had a higher risk of DEs, compared to good sleepers (odds ratio OR = 2.005; 95 % confidence interval CI: 1.933–2.080; *P* < 0.001). Table 4 illustrates the relationship between the eight components of sleep and DEs. High bedtime resistance, delayed sleep onset, irregular sleep duration, sleep anxiety, night walking, parasomnia, sleep-disordered breathing, and daytime sleepiness were associated with a higher risk of DEs. However, after correcting for age, sex, BMI, and other risk factors, all ORs were slightly lower, but the association between sleep dimensions and DEs risk remained statistically significant (Figure 2).

**Table 3:** Logistic regression analysis between sleep components and dry eye syndromes.

CSHQ component	NDEs (*n* = 146,895)	DEs (*n* = 22,185)	DEs a		DEs b	
			OR (95 %CI)	P	OR (95 %CI)	P
Poor sleep(CSHQ>41)	94,129(64.08 %)	17,889(80.64 %)	2.383(2.301,2.469)	<0.001	2.005(1.933,2.080)	<0.001
Good sleep(CSHQ≤41)	52,766(35.92 %)	4296(19.36 %)				
Bedtime resistance	67,161(45.72 %)	12,038(54.26 %)	1.481(1.438,1.525)	<0.001	1.313(1.273,1.354)	<0.001
Sleep onset delay	93,800(63.86 %)	15,611(70.37 %)	1.385(1.343,1.429)	<0.001	1.279(1.238,1.322)	<0.001
Sleep duration	112,149(76.35 %)	18,744(84.49 %)	1.693(1.629,1.759)	<0.001	1.538(1.477,1.600)	<0.001
Sleep anxiety	70,218(47.80 %)	13,305(59.98 %)	1.740(1.689,1.792)	<0.001	1.508(1.462,1.555)	<0.001
Night wakings	23,633(16.09 %)	5440(24.52 %)	1.693(1.637,1.751)	<0.001	1.516(1.464,1.570)	<0.001
Parasomnias	43,280(29.46 %)	10,490(47.28 %)	2.158(2.097,2.221)	<0.001	1.815(1.762,1.870)	<0.001
Sleep disordered breathing	12,986(8.84 %)	3944(17.78 %)	2.207(2.123,2.295)	<0.001	1.876(1.801,1.954)	<0.001
Daytime sleepiness	134,064(91.67 %)	20,737(93.47 %)	1.372(1.297,1.451)	<0.001	1.329(1.254,1.409)	<0.001

a Corrected for age, sex.

b Corrected for age, sex, BMI and recent eye surgery history, myopia or hyperopia status, contact lens usage, duration of contact lens wear, daily electronic device usage time, gaming time, e-book reading and Internet course engagement time, television viewing time, outdoor exercise duration, and presence of anorexic or food-paranoid tendencies.

**Table 4:** Linear regression analysis between different dry eye symptoms and sleeping.

CSHQ scores	DEQ-5							
	Discomfort		Dryness		Watery eyes		Total	
	*β (*95 %CI)	*P*	*β (*95 %CI)	*P*	*β (*95 %CI)	*P*	*β (*95 %CI)	*P*
Poor sleep (CSHQ>41)	0.336(0.323,0.349)	<0.001	0.320(0.307,0.334)	<0.001	0.117(0.110,0.125)	<0.001	0.774(0.743,0.804)	<0.001
Bedtime resistance	0.205(0.192,0.218)	<0.001	0.177(0.164,0.191)	<0.001	0.076(0.069,0.083)	<0.001	0.459(0.430,0.488)	<0.001
Sleep onset delay	0.150(0.137,0.164)	<0.001	0.142(0.129,0.156)	<0.001	0.041(0.033,0.048)	<0.001	0.333(0.303,0.364)	<0.001
Sleep duration	0.245(0.230,0.260)	<0.001	0.219(0.204,0.235)	<0.001	0.105(0.097,0.114)	<0.001	0.570(0.536,0.604)	<0.001
Sleep anxiety	0.287(0.275,0.300)	<0.001	0.254(0.240,0.267)	<0.001	0.105(0.098,0.112)	<0.001	0.646(0.617,0.675)	<0.001
Night wakings	0.236(0.219,0.252)	<0.001	0.230(0.213,0.247)	<0.001	0.128(0.118,0.137)	<0.001	0.593(0.556,0.630)	<0.001
Parasomnias	0.363(0.350,0.377)	<0.001	0.348(0.334,0.362)	<0.001	0.160(0.153,0.168)	<0.001	0.871(0.841,0.902)	<0.001
Sleep disordered breathing	0.400(0.379,0.421)	<0.001	0.394(0.373,0.415)	<0.001	0.154(0.143,0.166)	<0.001	0.969(0.902,0.995)	<0.001
daytime sleepiness	0.200(0.178,0.222)	<0.001	0.181(0.158,0.204)	<0.001	0.056(0.043,0.068)	<0.001	0.437(0.387,0.488)	<0.001

Logistic regression analysis, stratified by age and sex, revealed that poor sleepers of all categories had a higher risk of DEs than their counterparts who were good sleepers (Table 5). The relationship between the CSHQ scores and the three DEQ-5 subscales (discomfort, dryness, and watery eyes) is shown in Table 4. There was a significant relationship between the three DE symptoms and sleep (after correcting for age, sex, BMI, and other risk factors).

**Table 5:** Analysis of association between dry eye syndromes and sleep stratified by age and gender.

Age	n	DEs (n, %)	Poor sleep (n, %)	DEs a	
				OR (95 %CI)	*P*
6	8541	1173(13.73 %)	6634(77.67 %)	2.158(1.778,2.619)	<0.001
7	15,525	1867(12.03 %)	11,149(71.81 %)	2.186(1.905,2.507)	<0.001
8	35,118	4327(12.32 %)	24,598(70.04 %)	2.089(1.914,2.279)	<0.001
9	28,024	3590(12.81 %)	18,875(67.35 %)	1.948(1.778,2.135)	<0.001
10	31,355	4180(13.33 %)	20,222(64.49 %)	1.943(1.790,2.110)	<0.001
11	27,985	3825(13.67 %)	16,966(60.63 %)	2.051(1.886,2.231)	<0.001
12	22,532	3223(14.30 %)	13,574(60.24 %)	1.877(1.717,2.052)	<0.001
Gender				DE b	
Male	88,003	12,216(13.88 %)	57,897(65.79 %)	1.953(1.860,2.050)	<0.001
Female	81,077	9969(12.30 %)	54,121(66.75 %)	2.074(1.962,2.192)	<0.001

a Corrected for sex, BMI and risk factors.

b Corrected for age, sex, BMI and recent eye surgery history, myopia or hyperopia status, contact lens usage, duration of contact lens wear, daily electronic device usage time, gaming time, e-book reading and Internet course engagement time, television viewing time, outdoor exercise duration, and presence of anorexic or food-paranoid tendencies.

Sleep duration and DEs were significantly associated (Table 6). The study categorized average sleep time into three groups, as follows: ≤ 9 h, 9–10 h, and > 10 h. Logistic regression analyses revealed that children sleeping 〈 10 h were more likely to have DE than those who slept 〉 10 h.

**Table 6:** Logistic regression analysis between sleeping time and dry eye syndromes.

Sleeping time	DEs a		DEs b	
	OR (95 %CI)	*P*	OR (95 %CI)	*P*
≤9h	1.666(1.575,1.762)	<0.001	1.532(1.446,1.632)	<0.001
9h-10h	1.244(1.176,1.315)	<0.001	1.236(1.167,1.310)	<0.001
>10h	-	-	-	-

a Corrected for age, sex.

b Corrected for age, sex, BMI and recent eye surgery history, myopia or hyperopia status, contact lens usage, duration of contact lens wear, daily electronic device usage time, gaming time, e-book reading and Internet course engagement time, television viewing time, outdoor exercise duration, and presence of anorexic or food-paranoid tendencies.

## Discussion

To our knowledge, this study is the first to examine the relationship between DEs and sleep in a large sample of children in China. Of the 169,080 children included in this study, 13.12 % had DEs, and 66.25 % had poor sleep. A study of DE in India, including 47,260 children aged < 10 years, showed a 0.33 % prevalence [11]. A Korean study of children aged 6–12 years showed a 6.6 % prevalence of DE [12]. The prevalence of DE in children in China is higher than that in other countries, ranging from 13 % to 28 % [13,14].

There are several possible reasons for the differences in DE prevalence. First, as there are no specific diagnostic criteria for DE in children, the different diagnostic criteria chosen by the studies may have affected the reported prevalence rates. In the present study, the DEQ-5 questionnaire was used to diagnose DEs in children, whereas studies in Korea and India have used symptoms and signs to diagnose DE, following protocols similar to those used for adults. Second, the clinical symptoms of DE in children and adults are different, with adults presenting with obvious and easy-to-detect DE. In children, the most common symptoms of DE include an increase in the number of transient eye movements and visual fatigue, with children having a limited ability to communicate their experience. Therefore, DE is easy to miss or misdiagnose in the clinic, leading to an underestimation of the incidence rate. Meanwhile, the prevalence of DE has been shown to differ among regions and ethnic groups, with the TFOS DEWS II study identifying individuals of Asian descent as having an increased risk for DE [15]. A Chinese study showed a DE prevalence similar to the present findings.

The present study found that 80.64 % of the children with DEs had poor sleep. Children with poor sleep quality were more likely to have DE compared to their counterparts with good sleep quality, and this relationship persisted after correcting for age, sex, BMI, and other risk factors. In addition, this study found an association between DE and sleep in children of all ages and sexes. In a population-based study conducted in China (*n* = 2830), Yu et al. [16] found that patients with DE were more likely than the unaffected population to have sleep disturbances. The authors used the Pittsburgh Sleep Quality Index (PSQI) and the Ocular Surface Disease Index (OSDI) to assess DE symptoms and found an association between the total OSDI score and six of the seven components of the PSQI. Only sleep medication use was not significantly associated with the total OSDI score. Ong et al. [17] conducted a study on 120 United States Army veterans and concluded that sleep apnea was a significant risk factor for DE. Kaido et al. [18] conducted a study on 383 Japanese office workers and found that 45 % of patients with DE had sleep disorders and that the Schirmer score, DE symptom, and PSQI scores were significantly correlated. Zhang et al. [19] conducted a study on 1902 Chinese high school students and found that poor sleep quality was an important risk factor for DE. In the present study, the authors used the CSHQ questionnaire, which is more suitable for assessing children's sleep in terms of eight components, as follows: bedtime resistance, sleep onset delay, sleep duration, sleep anxiety, night wakings, parasomnias, sleep-disordered breathing, and daytime sleepiness. The DEQ-5 is also more suitable than other methods for evaluating DE in children [9]. The authors found significant correlations between DE and all sleep parameters in children. Children with sleep-disordered breathing were more likely to have a DE. Children with sleep-disordered breathing may experience intermittent hypoxia, which triggers a chronic systemic inflammatory state and immune alterations [20]. Elevated levels of pro-inflammatory cytokines, such as interleukin (IL)-1, IL-6, IL-8, tumor necrosis factor-α (TNF-α), and Fas receptor-positive lymphocytes, are associated with this process. Inflammation also plays a key role in DE, with increased tear film osmolarity and inflammation leading to ocular surface damage [21]. Lee et al. [22] has demonstrated that acute sleep deprivation rapidly compromises ocular surface health by worsening tear film stability, increasing osmolarity, and suppressing tear secretion. This is consistent with experimental findings in mice [23] where sleep deprivation not only reduced tear secretion but also led to cumulative corneal epithelial damage over a 10-day period. The observation that these changes recovered to near-normal levels after a 14-day rest period underscores the potential reversibility of sleep deprivation-induced ocular surface dysfunction.

This study showed that children who slept < 10 h were more likely to have DEs than those who slept for > 10 h. In a Korean study [24] of adults (*n* = 15,878), the OR of DE was 1.20 (95 % CI: 1.05–1.36) for short sleep duration (< 5 h per night) compared with optimal sleep duration (6–8 h). There was no significant difference in the prevalence of DE between long sleep duration (≥ 9 h) and optimal sleep duration. The results of a Singaporean study by Lim et al. [25] demonstrated that both poor sleep quality and short sleep duration (< 5 h) were significantly and independently associated with DE. The odds of experiencing DE were 1.73 times higher (95 % CI: 1.17–3.57) among individuals with less than 5 h of sleep compared to those with 5–9 h of sleep duration. The recommended optimal amount of sleep for children is 9 h or more, and this study found that children who slept fewer hours were more likely to develop DE. This may be because people who sleep for less time keep their eyes open longer. Consequently, the amount of eye exposure to detrimental environmental conditions may be increased. A previous study reported [26] that prolonged ocular exposure decreases blinking frequency, leading to DE symptoms.

The relationship between DE and sleep remains unclear; however, several potential mechanisms have been described. First, sleep disorders increase the levels of stress hormones, including cortisol, epinephrine, and norepinephrine, which depress the parasympathetic nervous system and increase sympathetic tone, leading to decreased tear production [27,28]. Secondly, sleep disorders can lead to excessive diuresis and natriuresis. Although antidiuretic hormone levels remain unchanged by sleep disorders, the circadian rhythm of the renin-angiotensin-aldosterone system is significantly altered. Body dehydration associated with sleep disorders may be due to a decrease in blood pressure during the night, as well as a decrease in the levels of the renin-angiotensin-aldosterone system activity [29]. Aldosterone acts on the kidneys, and its main role is to promote water reabsorption; a decrease in aldosterone leads to a decrease in water reabsorption and, consequently, a decrease in tear volume. Altered levels and overproduction of these hormones can cause the body to be in a state of relative dehydration, which affects tear production. In addition, sleep deprivation induces elevated expression of several inflammatory factors, such as C-reactive protein, IL-6 and IL-1. Inflammation also plays a key role in the pathogenesis of DE. Many inflammatory factors, such as IL-6, IL-1, and TNF-α, are elevated in the tear fluid of patients with DE compared with those in patients without DE. These inflammatory factors cause the release of acetylcholine and norepinephrine, which decreases tear secretion [30].

## Strengths and limitations

This study presented the most extensive pooled analysis of studies investigating the link between DEs and sleep in Chinese children. This comprehensive approach allowed us to establish a robust association between DEs and sleep among children in Tianjin, China, with high confidence. In addition, this study adjusted for important confounders in the association between sleep duration and DE. The study investigated the relationship between DEs and sleep, using a large sample, making the findings generalizable.

However, this study had some limitations. First, both DE incidence and sleep quality were assessed cross-sectionally, precluding discussions of causality. Second, the participants were not screened for signs of DE at the time of participation in the study. This study relied only on symptom-based questionnaires. Given the well-documented discordance between DE symptoms and clinical signs, examining ocular surface indicators of DE could offer valuable insights. Therefore, this study can only demonstrate the relationship between dry eye symptoms and sleep in children. Additionally, this study analyzed data based on self-reported and parent-reported questionnaires, which may introduce random measurement errors. Such measurement errors typically bias toward the null for linear relations, meaning observed associations likely underestimate the true relationship. Third, this study was initially designed with a primary focus on behavioral risk factors for DEs, the adjustment for confounding variables was partial, leaving several risk factors for DE and poor sleep unaccounted for, including history of allergic conjunctivitis, atopy, meibomian gland dysfunction, blepharitis, systemic diseases linked to DE (as rosacea), vitamin A intake, childhood cognitive function, and emotional problems, which may lead to residual confounding that could affect the odds ratios (ORs) reported in this study.

In conclusion, in this cross-sectional investigation encompassing children aged 6–12 years, DEs affected 13.12 % of participants, whereas 66.25 % reported poor sleep. The prevalence of poor sleep was 80.64 % (17,889/22,185) in children with DES, compared to 64.08 % (94,129/146,895) in those without DEs. Additionally, all domains of sleep were significantly poorer in participants with dry eye symptoms in children, and these associations remained significant after adjusting for comorbidities.

Notably, Ophthalmologists should remain vigilant about monitoring sleep-related issues when treating pediatric DE cases. Furthermore, prospective cohort studies are warranted to evaluate the causal links between DE and sleep, potentially revealing whether sleep interventions can alleviate DE symptoms.

## Clinical trial registration number

ChiCTR2200065710; 2022-11-13.

## Funding

This work was supported by the National Natural Science Foundation of China under Grant 82070929, Sponsored by Tianjin Health Research Project under Grant TJWJ2024QN027 and by the Tianjin Key Medical Discipline Construction Projectunder Grant TJYXZDXK-3–004A-2.

## Authors' contributions

All authors contributed to the study conception and design. QH, ZWS and RXL collected the data. QH, ZWS and RXL analyzed the data and wrote the manuscript. QH, ZWS and RXL contributed equally to this work and are both considered first authors. RHW and LL verified the analytical methods, provided critical feedback, and helped revise the final manuscript. All authors have read and approved the final manuscript. The authors would like to thank Editage (www.editage.cn) for English language editing.

## Data availability statement

The raw data supporting the conclusions of this article will be made available by the authors, without undue reservation.

## Ethics approval and consent to participate

Ethical approval for this study was obtained from the Medical Ethics Committee of Tianjin Medical University Eye Hospital. The study procedures adhered to the tenets of the Declaration of Helsinki. Informed consent has been obtained from the students' parents in this study.

## Consent for publication

Not applicable.

## Availability of data and materials

All data generated or analyzed during this study are included in this published article.

## Conflicts of interest

The authors declare no conflicts of interest.

## Appendix Supplementary materials

Image, application 1
