# Supplementary material for: Unraveling the relationship between childhood dry eye symptoms and sleep patterns
Source: J Pediatr (Rio J). 2025 Nov 11;102(1):101471. doi: 10.1016/j.jped.2025.101471 (PMC12651506; doi:10.1016/j.jped.2025.101471)
Supplement: Supplementary file 1 [file mmc1.docx]

**JPED-D-25-00288_ Supplementary Material**

**Supplementary Material 1. The letter to parents**

Dear students and parents of students:

Greetings!

Children and adolescents are in the stage of physical and mental development, and dry eye is a common ocular surface disease, which not only seriously affects learning, but also can cause psychological and behavioral. Problems. Recently, the incidence of DE has shown a decreasing trend with age, with an increasing prevalence in children and adolescents. The treatment of DE imposes a huge economic burden on society and patients. DE treatment costs more than $50 billion annually in the United States. It is critical to identify risk factors for DE that can be improved. With continued research on DE risk factors, lifestyle-related DE has been included as a new type of DE. Lifestyle-related DE risk factors in children may include video display terminals, outdoor activities, vitamin A deficiency, and poor sleep. Sleep problems in children are a global public health concern. Research has linked adequate sleep to better child performance, behavior, cognition, emotional regulation, and physical health. The American Academy of Sleep Medicine and Sleep Research expert consensus suggests that the recommended amount of sleep for adults and adolescents should be >7 and 9 hours, respectively. Protecting the eye health of students has become a key issue for the government and the society in terms of youth health. Tianjin Medical University Eye Hospital will conduct an epidemiological survey and monitoring of the eye health of primary school students in Tianjin. We will carry out a comprehensive and detailed monitoring and survey, through the "society-school-family" to take care of children's eyes, so that they can have a bright future.

Before you decide whether or not to take part in this study, please read the following as carefully as possible, as it will help you to understand the study and why it is being carried out, as well as the benefits, risks and discomforts you may experience if you take part. If you wish, you can also discuss this with your relatives and friends to help you make your decision. This study was approved by the Medical Ethics Committee of the Tianjin Medical University Eye Hospital (ChiCTR2200065710). The procedures used in this study adhered to the tenets of the Declaration of Helsinki.

1.What you will need to do if you take part in the study

You will be asked to sign an informed consent form.

You will be asked to fill in the questionnaire as appropriate.

2. Possible benefits of participating in the study

This survey may help to analyse the children's eye surface condition and sleep, and to protect the eye health and mental health of children and adolescents.

3. Possible adverse reactions, risks and discomfort, inconvenience of participating in the study

We will ensure the confidentiality of your and your child's information in this survey and

will never disclose your or your child's information.

4. Related Costs

There is no cost to you or your child to participate in this survey.

5. Is personal information confidential?

All statistical analyses will not have your and your child's name or any information about them. The confidentiality of all records will be held to the highest degree possible in accordance with the law.

6. How can I get more information?

Participate in this questionnaire and if you have any questions, please contact the Ethics Committee Office of Tianjin Medical University Eye Hospital.

7. You can voluntarily choose to participate in the study and withdraw from the study in the middle of the study

Whether or not to take part in the survey is entirely up to you and your child. You can also refuse to take part in the study, and this will not affect your or your child's rights and interests.

8. What should I do now?

It is up to you to decide whether or not to take part in this survey. You can discuss your decision with your family or friends.

**Supplementary Material 2**

**2.1** If your child has visited the hospital in the past month due to a severe eye infection or inflammation, or has suffered from eye trauma, chemical burns to the cornea or conjunctiva, you do not need to fill out this questionnaire.

Yes (Exit the questionnaire) No (Continue filling out the questionnaire)

**2.2** **Personal Information and Lifestyle Habits Questionnaire**

Name： Gender： Date of Birth(year/month)： Height and Weight (cm/kg):：

Have you had any eye surgery?

Hyperopia (farsightedness) prescription

Myopia (nearsightedness) prescription

Do you wear contact lenses?

How long have you been wearing contact lenses?

Total daily screen time

Daily screen time spent gaming

Daily screen time spent reading e-books

Daily screen time spent attending online classes

Daily TV viewing time

Total daily outdoor activity time

Do you have anorexia or picky eating habits?

**2.3 5-Item Dry Eye Questionnaire (DEQ-5)**

**1.Questions about EYE DISCOMFORT:**

1. During a typical day in the past month, how often did your eyes feel discomfort?

0 Never

1 Rarely

2 Sometimes

3 Frequently

4 Constantly

b. When your eyes felt discomfort, how intense was this feeling of discomfort at the end of the day, within two hours of going to bed?

Never Not at All Very

have it Intense

0 1 2 3 4 5

**2.Questions about EYE DRYNESS:**

a. During a typical day in the past month, how often did your eyes feel dry?

0 Never

1 Rarely

2 Sometimes

3 Frequently

4 Constantly

b. When your eyes felt dry, how intense was this feeling of dryness at the end of the day, within two hours of going to bed?

Never Not at All Very

have it Intense Intense

0 1 2 3 4 5

**3.Question about WATERY EYES:**

During a typical day in the past month, how often did your eyes look or feel excessively watery?

0 Never

1 Rarely

2 Sometimes

3 Frequently

4 Constantly

**2.4 Children's Sleep Habits Questionnaire (CSHQ)**

parents were asked to recall sleep behaviors occurring over a “typical” recent week. Items are rated on a three-point scale: “usually” if the sleep behavior occurred five to seven times/week; “sometimes” for two to four times/week; and “rarely” for zero to one time/week. Some items were reversed in order to consistently make a higher score indicative of more disturbed sleep.

Weekday sleep hours:

Weekend sleep hours:

1. Bedtime Resistance usually sometimes rarely

Goes to bed at same time

Falls asleep in own bed

Falls asleep in other's bed

Needs parent in room to sleep

Struggles at bedtime

Afraid of sleeping alone

2.Sleep Onset Delay usually sometimes rarely

Falls asleep in 20 minutes

3. Sleep Duration usually sometimes rarely

Sleeps too little

Sleeps the right amount

Sleeps same amount each day

4. Sleep Anxiety usually sometimes rarely

Needs parent in room to sleep

Afraid of sleeping in the dark

Afraid of sleeping alone

Trouble sleeping away

5. Night Wakings usually sometimes rarely

Moves to other's bed in night

Awakes once during night

Awakes more than once

6.Parasomnias usually sometimes rarely

Wets the bed at night

Talks during sleep

Restless and moves a lot

Sleepwalks

Grinds teeth during sleep

Awakens screaming, sweating

Alarmed by scary dream

7. Sleep Disordered Breathing usually sometimes rarely

Snores loudly

Stops breathing

Snorts and gasps

8. Daytime Sleepiness usually sometimes rarely

Wakes by himself

Wakes up in negative mood

Others wake child

Hard time getting out of bed

Takes long time to be alert

Seems tired

Watching TV

Riding in car
